# Prevalence and risk factors for cage subsidence after lumbar interbody fusion: A protocol for systematic review and meta-analysis

**DOI:** 10.1097/MD.0000000000028085

**Published:** 2021-12-10

**Authors:** Qiujiang Li, Xingxia Long, Lin Shi, Yinbin Wang, Tao Guan, Jinhan Lv, Lijun Cai

**Affiliations:** a Ningxia Medical University, Yinchuan, Ningxia, China; b Department of Orthopedics, People's Hospital of Ningxia Hui Autonomous Region, Yinchuan, Ningxia, China; c West China Hospital, Sichuan University, Sichuan, China; d Traditional Chinese Medicine Hospital Dianjiang Chongqing, Chongqing, China.

**Keywords:** cage subsidence, lumbar interbody fusion, meta-analysis, risk factors, systematic review

## Abstract

**Introduction:**

Lumbar interbody fusion (LIF) is an effective treatment for lumbar degenerative diseases. Cage subsidence (CS) contitutes one of the most common postoperative complications. Many risk factors for CS after LIF have been reported in some studies. However, controversies still exist. The objective of this study will be to summarize data on the prevalence and risk factors of CS after LIF.

**Methods and analysis:**

Our study present a protocol that conducted a systematic review and meta-analysis of prevalence and risk factors for CS after LIF. Two reviewers retrieved the relevant articles using the 5 databases (PubMed, Scopus, EMBASE, Cochrane Library, and Web of Science) from inception to May 31st, 2021. Primary outcome will be the prevalence of CS after LIF. Second outcomes include the risk factors associated with postoperative CS and clinical outcomes associated with postoperative CS. Three reviewers will screen citation titles and abstracts and evaluated full-text of each potentially relevant citation, and then extracted the data using a data extraction form. Any discrepancies in decisions between reviewers will be resolved through discussion. We assessed the methodological quality and risk of bias of the included studies based on the Newcastle–Ottawa Quality Assessment Scale (NOS). The aim of the extra analysis is to explore the explanations of the heterogeneity (age, gender, race, year of publication, type of study and surgical procedure). Publication bias will be assessed by Begg test, Egger test and funnel plots.

**Ethics and dissemination:**

No primary data will be collected and individual patient information and endangering participant rights, thus ethics approval is not required. Findings will be reported through publication and media.

**Protocol registration number:**

PROSPERO CRD42021257981 (https://www.crd.york.ac.uk/PROSPERO/#joinuppage).

## 1 Introduction

Currently, lumbar interbody fusion (LIF) procedures have been widely used for the treatment of lumbar degenerative disease.^[1,2]^ LIF is a technique used for fixing the intervertebral joints and maintaining stability through the implantation of the lumbar interbody cage filled with autologous bones or allograft bones to facilitate the fusion of the 2 adjacent vertebrae.^[3,4]^ Meanwhile, It also restores the height of the intervertebral space and the curvature of the spine.^[5]^ However, LIF surgery runs the risk of complications, such as loosening/rupture of the internal fixation, adjacent segment degeneration, and cage migration.^[6–9]^ Cage subsidence (CS) is one of the most common complications of cage migration, which is attracting more and more worldwide attention.^[10,11]^ Currently, there are 2 different views regarding CS. Some scholars believe that CS after LIF is a long-term slow change that the bone grafts and vertebrae are fused together, which is the usual imaging findings postoperative.^[12,13]^ Mild CS is a normal phenomenon without causing any clinical symptoms.^[14]^ In contrast, some scholars believe CS depth may increase with prolonged follow-up, which may cause the reduction of segmental height and the loss of segmental lordosis, may lead nerve compression and correlated with adverse patient outcomes, such as recurrent low back pain and neurological symptoms, internal fixation failure and an increase in the reoperation rate.^[15,16]^

At present, the prevalence of CS varies from 15.9% to 70% in the literature.^[10]^ Several studies have reported CS is associated with many factors, including surgical segements, surgiacal levels, cage shape, cage position, cage material, bone mineral density.^[17–20]^ However, the scope was limited and the sample size was small in most of these studies, still there are controversies. The importance of risk factors for CS, consequently, is difficult to establish. More clinical data are needed to support the conclusion. Due to this, the key to reduce the prevalence of CS is how to establish the risk factors.

To our knowledge, there are no systematic reviews and meta-analyses to evaluate the prevalence and risk factors for CS after LIF. Our study contributes to clarify the risk factors of CS based on integrating the results of numerous clinical studies.

## 2 Methods

This protocol has been reported under the guidance of the Preferred Reporting Items for Systematic Reviews and Meta-analysis Protocols (PRISMA-P).^[21,22]^

### 2.1 Inclusion criteria

#### 2.1.1 Types of studies

Observational studies (including cohort and case-control studies) with available data on the prevalence and risk factors associated with CS among patients following LIF. If, in case that several articles from the same trial were published, the study that had the most relevant information or the longest follow-up period might be most appropriate. Case series, case reports, reviews, letters to the editor, comments and conference abstracts will be excluded.

#### 2.1.2 Types of patients

Inclusion criteria for study populations will be all patients undergoing LIF surgery during the study period and age older than 18 years.

#### 2.1.3 Types of outcome measures

1. The prevalence of CS after LIF.
2. The risk factors of CS after LIF.
3. The strength of correlation between each risk factor and CS.

### 2.2 Information sources and search strategy

The electronic search database includes PubMed, Scopus, EMBASE, Cochrane Library and Web of Science. The dates range were from database inception to May 31st, 2021. In addition, grey literature was also searched. If data in the study were insufficient or missing, we will first contact authors for further information. To include as many relevant studies as possible, we consulted with content experts. A draft search strategy for PubMed is provided in Table 1.

**Table 1:** Search strategy in PubMed database.

No	Search items
1	Observational study. Mesh.
2	Observational. ti.ab.
3	Observe. ti.ab.
4	Study. ti.ab.
5	1 or 2–4
6	Migration. ti.ab.
7	Subsidence. ti.ab.
8	6 or 7
9	Cage. ti.ab.
10	Cages. ti.ab.
11	9 or 10
12	Lumbar interbody fusion. ti.ab.
13	5 and 8 and 11 and 12

### 2.3 Study selection

**Figure 1:**
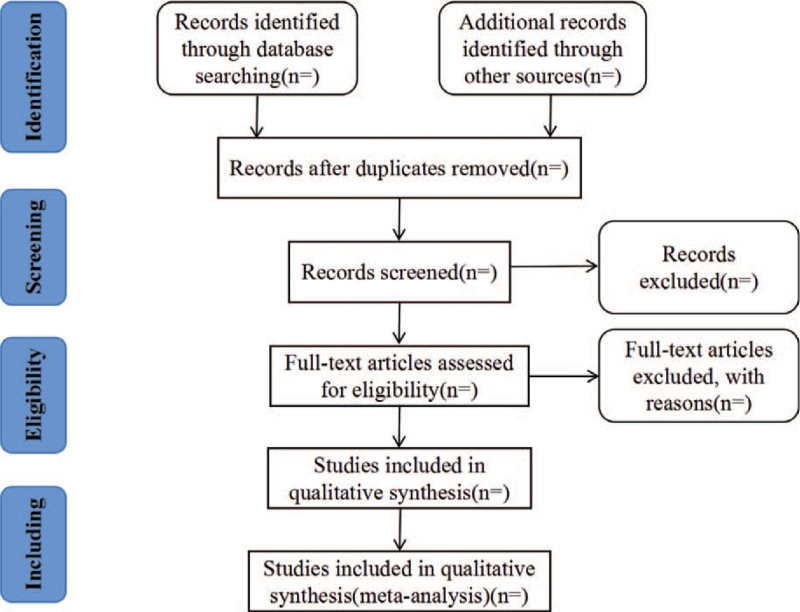
PRISMA flow diagram of the identification, screening and eligibility of included articles.

The literature was screened by two reviewers (QJ L and XX L) independently. Firstly, those literatures that clearly did not meet the inclusion criteria were excluded by reading the title and the abstract. Then, the selected articles that fulfilled the inclusion criteria will be read in their entirety. Finally, 2 reviewers (YB W and L S) will extract the data using a data extraction form. Two reviewers (JH L and T G) cross-checked on collected data. When the checked results fail to reach an agreement, arbitration will be resolved through discussion with a third reviewer (LJ C). If data were depicted in graphs or incomplete, we will try to contact authors for obtaing original data by email. The study screening process is shown in Figure 1.

### 2.4 Risk of bias assessment

The Newcastle–Ottawa quality assessment scale (NOS) is a validated tool to assess the quality of case-control and cohort studies.^[23]^ Two reviewers (QJ L and XX L) assessed the methodological quality and risk of bias of the included studies based on the NOS for cohort and case-control studies. According to the score of NOS, studies were divided into 3 categories of quality: high quality (score 7–9), medium quality (score 4–6), and low quality (score 0–3).^[24]^ When the results of methodological quality and risk of bias fail to reach an agreement, arbitration will be resolved through discussion with a third reviewer(JH L).

### 2.5 Statistical collection and analysis

#### 2.5.1 Data extraction

Study independent data extraction was done by 2 authors (YB W and L S). Planned data extraction included: the name of the first author, year of publication, country or region, study types, sex, age, the total number of disc and patients, the diagnosis criteria of cage subsidence, the prevalence of CS, follow-up time and other related risk factors. Factors that may affect CS risk include sex, age, preoperative diagnosis, surgical segments, surgical levels, cage shape, cage position, cage material, bone mineral density, surgical procedure. When data extraction fail to reach an agreement, arbitration will be resolved through discussion with a third reviewer (JH L). If data were depicted in graphs or incomplete, we will try to contact authors for obtaing original data by email.

#### 2.5.2 Assessment of heterogeneity

Heterogeneity was assessed by means of the *I*^2^ statistic. If *I*^2^ ≤ 50%, fixed-effects model was used; if not, random-effects model was used. The source of any observed heterogeneity was identified according to remove the high heterogeneity factors one by one in our study.

#### 2.5.3 Data synthesis

Prevalence estimate of CS after LIF will be presented as 95% confidence intervals. Relative risk ratios or odds ratios with 95% confidence intervals will be used to assess the strength of correlation between each risk factor and CS. If there is minor heterogeneity or the source could be found, although heterogeneity is high, a meta-analysis will be performed. Otherwise, only a narrative synthesis will be carried out. When there is significant heterogeneity, we will perform a subgroup analysis or meta-regression analysis. After data were synthesized, analysis was performed using RevMan v.5.3 Software provided by the Cochrane Collaboration.

#### 2.5.4 Subgroup analysis

In order to reduce any observed heterogeneity effect on the outputs of the meta-analysis, subgroup analysis will be carried out based on country or region, preoperative diagnosis, surgical segments, surgical levels, surgical procedure and follow-up time (short-term or long-term), when sufficient data are available.

#### 2.5.5 Meta-regression analysis

Meta-regression analysis will be performed to explore whether study characteristics had a relationship with the effect sizes using study characteristics (country or region, preoperative diagnosis, surgical segments, surgical levels, surgical procedure and follow-up time) as covariates.

#### 2.5.6 Assessment of reporting biases

We evaluate the reporting bias by funnel plot. To avoid the effect of subjective visual observation on funnel plot, Begg and Egger tests will be carried out to assess the asymmetry of funnel plot, as supplementary approach.

#### 2.5.7 Quality control of the systematic review and meta-analysis

The quality control of evidence was assessed with Grading of Recommendations Assessment, Development, and Evaluation system^[25]^ based on the 5 evaluation items include risk of bias, inconsistency, indirectness, imprecision and publication bias. The results of evidence quality will be divided into 4 levels: very low, low, medium, high.

#### 2.5.8 Patient and public involvement

Patients and the public were not involved in the design or planning of this protocol for systematic review and meta-analysis.

## 3 Discussion

CS is one of the most common postoperative complications in lumbar interbody fusion, which is correlated with adverse patient outcomes, such as recurrent low back pain and neurological symptoms, internal fixation failure and an increase in the reoperation rate.^[10,11,26]^ Therefore, identification of risk factors for CS after LIF will provide best practices for the assessment of risk factors and postsurgical preventive/clinical interventions for CS. A systematic review and meta-analysis presented in our protocol will identify, collect, evaluate and integrate the existing knowledge in the risk factors and prevalence of CS. To our knowledge, this will be the first study to summarize the literature on the risk factors and prevalence associated with CS. The manuscript will be structured using the reporting guidance provided in the PRISMA statement.^[22]^ The results of this study will provide evidence for clinicians and further guide researchers to embark on relevant studies, especially in a high-risk cohort for developing CS. Although our study screened the literatures according to the strict inclusion and exclusion criteria, heterogeneity is still expected and evaluated. The prevalence of CS was reported to be 15.9% to 70%^[10]^ and the risk factors of CS were reported inconsistently among different studies, it is a major limitation of the study. Finally, we performed subgroup analysis and meta-regression analysis to decrease the heterogeneity. The results of our study will be published on an international peer-reviewed journal and disseminated to the public through publications, conferences, and meetings.

## Author contributions

**Conceptualization:** Qiujiang Li, Xingxia Long, Lin Shi, Lijun Cai.

**Data curation:** Qiujiang Li, Xingxia Long, Lin Shi, Yinbin Wang, Tao Guan, Jinhan Lv.

**Funding acquisition:** Jinhan Lv, Lijun Cai.

**Investigation:** Lijun Cai.

**Methodology:** Lijun Cai.

**Writing – original draft:** Qiujiang Li, Xingxia Long, Yinbin Wang.

**Writing – review & editing:** Qiujiang Li, Lijun Cai.
